# Validation of a Newly Developed Instrument Establishing Links Between Motivation and Academic Hardiness

**DOI:** 10.5964/ejop.v12i1.997

**Published:** 2016-02-29

**Authors:** Spiridon Kamtsios, Evangelia Karagiannopoulou

**Affiliations:** a Independent Researcher, Ioannina, Greece; b Department of Philosophy, Pedagogy and Psychology, Section Psychology, University of Ioannina, Ioannina, Greece; Academy of Special Education, Warsaw, Poland

**Keywords:** academic hardiness, elementary school children, validation, convergent validity

## Abstract

The purpose of the study was to establish the reliability, the structural and the convergent validity of the “Dimensions of Academic Hardiness Questionnaire” for late elementary school children. A sample of children (N = 1264) aged 10-12 years completed the questionnaire and the “Athens Coping Scale”. Multiple fit indices provided support that the 9-factor model had a good fit to the data. Reliability coefficients ranged from .68 to .83. The study provided also preliminary evidence of convergent validity of the “Dimensions of Academic Hardiness” scores with one theoretically related measure, the “Athens Coping Scale”. The results enrich the notion of Academic Hardiness in late elementary school children as the role of awareness and the role of children’s previous experiences has been distinguished. The relation between the “Dimensions of Academic Hardiness” and achievement goal orientations in children learning is also noted. These findings are discussed in the context of the relevant literature.

## Introduction

Hardiness is well established as a pattern of attitudes and skills that helps one remain resilient under stress (Maddi, 2006). Hardiness has been theorized to have a buffering effect on stress and to influence the types of coping strategies utilized in response to the appraisal of stress (Maddi, Harvey, Khoshaba, Fazel, & Resurreccion, 2012). According to Maddi and Kobasa (1984), hardiness consists of commitment (vs. alienation), control (vs. powerlessness), and challenge (vs. threat). Persons strong in commitment believe that they can find something in whatever is going on that seems interesting or important. They are unlikely to engage in denial or feel disengaged. Persons strong in control believe they can beneficially influence outcomes through effort and they are unlikely to feel powerless. Those strong in challenge believe that life is best when they continue to grow in wisdom through learning from experiences, whether positive or negative. They are unlikely to expect uninterrupted comfort and security (Maddi, 2005). It is the interactive combination of commitment, control, and challenge that defines hardiness as the existential courage to face stressful circumstances openly and directly, and the motivation to do the hard work of dealing with them constructively (Maddi, 2006; Maddi et al., 2012).

Several studies indicated hardiness as a predictor of effective coping (Chan, 2000; Clarke, 1995; Florian, Mikulincer, & Taubman, 1995; Lambert, Lambert, & Yamase, 2003; Paleologou & Dellaporta, 2009). It has been proposed that high-hardy individuals engage in approach coping styles^i^ (problem focused coping strategies) for the purpose of transforming stressful events into situations that seem to be more manageable. Hardy attitudes lead to maintenance and enhancement of performance under stress. In contrast, low-hardy individuals tend to engage in avoidance coping styles, such as cognitive and behavioral disengagement and denial, to deal with a stressful situation (Maddi, 2006; Sheard, 2009).

Given evidence that psychological hardiness helps insulate individuals from the effects of stress, questions naturally arise regarding its generalizability across contexts and its influence on outcomes other than health (Cole, Field, & Harris, 2004). For Benishek and Lopez (2001), the initial question was: What might be the positive impact of hardiness in academic settings? To answer this question they suggested that two cognitively oriented theories, Kobasa’s (1979) hardiness theory and Dweck’s (2000) theory of academic motivation might be useful in understanding why some children persevere when faced with academic difficulties. They suggested the notion of Academic Hardiness, as their hypothesis was that children’s learning motivation and academic hardiness interact to predict children’s effective outcomes (Benishek, Feldman, Shipon, Mecham, & Lopez, 2005; Cole et al., 2004) and this interaction can increase understanding of the learning experience (Cole et al., 2004). Commitment was defined as students’ reported willingness to expend consistent effort and to engage in personal sacrifices in order to achieve academic excellence, irrespective of the content or demands of individual courses, instructors or personal interests. Challenge was defined as the students’ purposeful efforts to seek out difficult academic coursework and experiences and to justify such actions as inherently for personal learning. Control was defined as students’ beliefs that they possessed the capacity to achieve desired educational outcomes from personal effort and through effective emotional self-regulation in the face of academic stresses and disappointments (Benishek et al., 2005).

This conceptualisation guided to the development of the Academic Hardiness Scale (Benishek & Lopez, 2001; Benishek et al., 2005), reflecting commitment, challenge and control. The academic hardiness scale has been used in researchers with high school students and undergraduates without exploring the factorial validity of the scale (Golightly, 2007; Karimi & Venkatesan, 2009; Kinder, 2008). Some other studies reported psychometric weakness of the scale (Golightly, 2007; Kamtsios & Karagiannopoulou, 2011).

Although many studies with adults (Maddi, Harvey, Khoshaba, Fazel, & Resurreccion, 2009) and undergraduates (Hystad, Eid, Laberg, Johnsen, & Bartone, 2009; Mathis & Lecci, 1999) have shown that hardiness and academic hardiness provide a buffering effect to life-work stress and school-college stress respectively, there is no research in looking at the enhancement of hardiness and academic hardiness in elementary school children (Kamtsios & Karagiannopoulou, 2011, 2013a) in which school and achievement is a major source of stress (Compas, Connor-Smith, Saltzman, Thomsen, & Wadsworth, 2001; Nelms, 1999), even if they do not have examinations and weekly test as high school and senior high school children (Kamtsios & Diggelidis, 2008). Also, there has been little work on hardiness in high school students, although the hardiness construct seems useful in assisting school students in dealing with school-related stressors (Green, Grant, & Rynsaard, 2007). Besides there are no validated scales measuring exactly academic hardiness components (Benishek et al., 2005 mentioned that there are additional aspects of the academic hardiness construct that have yet to be identified), although the need to understand and examine the construct of hardiness and academic hardiness in different life stages, on different groups and in different cultural settings has been increasingly recognized (Benishek et al., 2005; Chan, 2000; Green et al., 2007; Kamtsios & Karagiannopoulou, 2013a), and although hardiness construct seems useful in assisting students in dealing with school-related stressors (Green et al., 2007). Some scales are not validated in different age samples and cultures (Benishek et al., 2005), whereas some others were modified with some wording changes to reflect a school situation rather than a work situation (Morrissey & Hannah, 1987).

Kamtsios and Karagiannopoulou (2011, 2013a) mentioned this potential gap in our knowledge regarding concepts related with academic hardiness in education. They believe that, as the school environment is particularly complex, there may be other variables associated with children’s academic hardiness and children’s commitment, control and challenge. In their first study (Kamtsios & Karagiannopoulou, 2013a) they explored the possible relevant aspects of academic hardiness and its components (commitment, control, and challenge), using qualitative methodology. Their aim was to develop a broader conceptualization of academic hardiness through the children’s perspective. They look specifically at children’s perceptions and experiences concerning the way they cope with a school failure in terms of commitment, control and challenge. Their findings bring into question additional aspects of academic hardiness and its components in primary school children (10-12 years) that have not been identified in the past and provided information to better understand the educational dynamics of academic hardiness constructs.

In their second study (Kamtsios & Karagiannopoulou, 2013b), drawing from the interviews, they developed a battery of items and they created the “Dimensions of Academic Hardiness Questionnaire”. After a set of exploratory factor analyses, the confirmatory factor analysis results provided support for the 9-factor solution (36 items) which explained 55.15% of the total variance. Scale scores showed adequate internal consistency and 2-week test-retest reliability. The findings were supportive to the academic hardiness theory as each factor corresponded conceptually to one of the three characteristics of the original academic hardiness theory (commitment, control, challenge). Their results also confirm and strengthen the relation between hardiness and achievement goal orientation in children learning. The nine factors which emerged (see Table 1) reflect the different ways in which late elementary school children try to cope with the school failure.

We believe in the importance to further examine the dimensions of academic hardiness. An integral part of the conceptualization of academic hardiness and its dimensions is that the educational circumstances (academic achievement, learning, school environment), for many children attending school, are inherently stressful (Compas et al., 2001; Pincus & Friedman, 2004) and this stress may make achievement and learning more difficult. The dimensions of academic hardiness, mentioned by Kamtsios and Karagiannopoulou (2013a) and evaluated in their second study by the “Dimensions of Academic Hardiness Questionnaire” for late elementary school children (Kamtsios & Karagiannopoulou, 2013b), may lead to hardy academic attitudes, which may influence how children experience and cope with stressful academic and school circumstances.

The present study presents the third part of an ongoing project aiming to develop an academic hardiness questionnaire for late elementary school children. This study reports the validation of the “Dimensions of Academic Hardiness Questionnaire” for late elementary school children (Kamtsios & Karagiannopoulou, 2013b) in a sample of children aged 10-12 years. The hypotheses guiding this study are as follows: First, we expected to confirm the factorial structure of the questionnaire in a new sample, using confirmatory factor analysis (CFA). Second, children’s coping style and the “Dimensions of Academic Hardiness” score are expected to be correlated. Specifically, it is expected that the total score of the “Dimensions of Academic Hardiness Questionnaire” will be statistically positively correlated with “approach” coping styles (problem focused coping strategies) whereas there will be no statistically significant correlation between the dimensions of academic hardiness and avoidance coping styles. Also, we expect that high academic hardiness children will engage in approach oriented coping strategies, such as assistance seeking, family support, problem solving and revision-reorganization, whereas low academic hardiness children will engage in avoidance oriented coping strategies such as avoidance, giving up – distancing and isolation.

Finally, the relation between the “Academic Hardiness Dimensions” and achievement goal orientations in children learning (task or performance orientation) is expected to be statistically established through a second order factor analysis indicating that motivation is underlying the dimensions of academic hardiness. Such a relation will support the content validity of the scale that joins hardiness and motivation.

## Method

### Sample

The participants of the study were 1264 children (647 boys and 617 girls) from 25 elementary public schools in northwest Greece. Grade 5 (580 children-45.9%) and grade 6 (684 children-54.1%) children took part in the study. Participants’ age ranged from 10 to 12 years. The schools and the children were randomly assigned to contribute to the research.

### Procedure

Children and their parents were provided with a letter informing them about the purpose of the study. The questionnaires were administered in the classroom to pupils who returned a signed parent consent form. An initial explanation concerning the research and instructions on how to answer the instrument was presented to the children. After the opportunity for clarification and questions, they responded to the measures. The completion of the questionnaires required 20-25 min. This phase of the study was conducted with the permission of the Greek Ministry of Education and the children voluntarily chose to participate.

### Measures

The children questionnaires used in this study were the following:

The “Dimensions of Academic Hardiness Questionnaire” for late elementary school children (Kamtsios & Karagiannopoulou, 2013b). The questionnaire represents a full range of children’s experiences, energies and actions in order to cope with a school failure. The questionnaire consists of 36 items allocated into 9 factors. The item development for the questionnaire was based on the analysis of interview data (for details see: Kamtsios & Karagiannopoulou, 2013a). Examples of the items are presented in Table 1. Pupils responded on a 4-point Likert-type scale, ranging from 1 (strongly disagree) to 4 (strongly agree). There are no reversely coded items. A high score on the 4-option Likert-scale indicated that the aspect being assessed by the question was perceived to occur frequently by the children. The questionnaire has shown internal consistency ranging from .68 to .83.

**Table 1:** Factors of the “Dimensions of Academic Hardiness Questionnaire” for Late Elementary School Children, Description, Number of Items and Example Items

No	Factor	Description	Number of items	Example item
1	Commitment: *comparing oneself with peers and acceptance from peers.*	Indicate children’s commitment-comparison and acceptance by schoolmates.	4	“I do my best at school so as my marks to be higher than those of my classmates”
2	Control-awareness: *use of effective coping strategies*	Effectiveness of different strategies in order for the children to achieve mastery orientation goals	6	“When my performance at school is not good, I try to find ways to face the problem”
3	Commitment: *adults’ acceptance*	Items mentioned that children can recognize what have value and importance for other individuals and be commitment to achieve them	4	“I do my best at schoolwork to prove to my parents that I can make it”
4	Commitment: *knowledge utility*	Children’s commitment to study further recognizing the usefulness of knowledge the next years of school life	5	“I believe that everything taught and learnt at school now can be also used in both secondary and high school”
5	Control-awareness: *attempt to avoid unpleasant feelings*	Children’s control and awareness for effort with regard to avoid the unpleasant feelings after a school failure	5	“I try not to get a low mark to avoid feeling disappointment and shame”
6	Commitment: *regulating priority to learning vs. enjoyment*	Children’s priority of learning and commitment to academic tasks and time management.	3	“I try to finish my homework first before I spend time with my friends”
7	Challenge: *dealing positively with hard subjects*	Failure is perceived as an experience that leads them to put more effort on study	3	“I find interest in my school subjects even though they may be difficult”
8	Commitment: *looking for help contributing to learning*	Children’s attitude to seek support when they face difficulties or when they do not accomplish well with the school lessons	3	“When I have difficulties with my schoolwork I ask for my parents’ help”
9	Challenge: *dealing with failure in a constructive way*	Children’s attitude to insist on their effort even if they meet difficulties (e.g. low grade) in the learning process	3	“Getting a low mark makes me try harder in order to get a higher one the next time”

Coping was measured by the “Athens Coping Scale Questionnaire” (Besevegis, 1996, 2001), which comprises 32 items. The “Athens Coping Scale Questionnaire” served as a criterion measure to assess the convergent validity of the “Dimensions of Academic Hardiness Questionnaire” for late elementary school children. Scores from the two scales were hypothesized to be correlated because the two questionnaires are theoretically related. The “Athens Coping Scale Questionnaire” has been especially designed for children aged 10 and above (Besevegis, 2001). The questionnaire has been used widely in studies (in Greece) with children and adolescents and its factorial validity has been confirmed (Amponi-Tsoura, 2006; Argyropoulou, Sidiropoulou-Dimakakou, & Besevegis, 2007; Giavrimis, Konstantinou, & Hatzichristou, 2003). The questionnaire consists of seven coping strategies. Students were asked to indicate on a 4-point Likert-type scale ranging from 0 (never) to 3 (often), the extent to which they use each of the coping strategies: (a) assistance seeking (4 items: e.g., “I asked somebody to help me out”), (b) family support (5 items: e.g., “I talked to my parents and asked their help”), (c) avoidance (6 items: e.g., “I started doing other things to keep myself busy and not think of what bothered me”), (d) giving up-distancing (5 items: e.g., “I decided that I could not do anything”), (e) isolation (4 items: e.g., “I shut myself off, I locked myself in my room”), (f) problem solving (4 items: e.g., “I increased my efforts in order to solve my problem”), (g) revision-reorganization (4 items: e.g., “I decided to change behavior in order to achieve the solution of the problem”). Assistance seeking, family support, problem solving and revision-reorganization are categorized in the approach coping styles, whereas avoidance, giving up – distancing and isolation are categorized in the avoidance coping styles (Besevegis, 2001).

### Data Analysis

Initially means and standard deviations were calculated for all factors. Confirmatory factor analysis (CFA) was used to test the structure of the 9-factor, 36-item “Dimensions of Academic Hardiness Questionnaire” scores, using EQS 6.1 statistical package (Bentler, 1995). A model was specified in which all items for each scale were allowed to load on the corresponding factor only. This measurement model was developed on the basis of the factor loadings from the exploratory and confirmatory analyses reported in a previous study (Kamtsios & Karagiannopoulou, 2013b).

Maximum likelihood (ML) estimation was used to address the possibility of non-normal distribution (Cantoni & Ronchetti, 2006) and to estimate the model parameters and the fit indices. ML has been found to produce more accurate fit indices and less biased parameters than generalized squares estimation (Olsson, Foss, Troye, & Howell, 2000). Hu and Bentler (1998) and Tanaka (1993) recommend examining multiple indices when evaluating the overall fit of a model in order to address these different aspects of fit.

The present study used a series of fit indices and error estimates to evaluate the adequacy of the measurement model on the basis of recommendations presented in the relevant literature (Hu & Bentler, 1999; Kline, 2000; Tanaka, 1993): the chi-square divided by the degrees of freedom (χ^2^/*df*), the comparative fit index (CFI), the goodness-of-fit index (GFI), the adjusted goodness-of-fit index (AGFI), the root mean square error of approximation (RMSEA), the root mean residual (RMR), the root mean square residual (RMSR), the normed fit index (NFI) and the non-normed fit index (NNFI). Values of the chi-square divided by degrees of freedom (χ^2^/*df*) that are less than 2 are commonly taken to indicate a good model fit (Hu & Bentler, 1998), and according to Kline’s (1998) recommendations, an acceptable proportion is 2:1 or 3:1. The CFI computes model fit by comparing the hypothesized model to a null model that assumes that are no relations among observed variables. GFI and AGFI are indexes of absolute fit. They provide a measure of variance or covariance that can be explained by the model, which is similar to a squared multiple correlation (Kline, 1998). AGFI adjusts the GFI by taking into account the number of estimated parameters in the model (Tabachnick & Fidell, 1996). Typically for these indices values > .90 indicate an acceptable fit to the data (Hu & Bentler, 1999; Kranzler & Keith, 1999). The RMSR is the square root of the mean of the squared discrepancies between the implied and the observed covariance matrices. The RMSEA is also based on the analysis of residuals and compensates for the effect of model complexity. A RMSEA value of less than .06 and a SRMR value of less than .08 indicate a good fit (Brown & Cudeck, 1993; Hu & Bentler, 1999).

We also used a second-order factor analysis as a tool to examine the regrouping of the nine factors into individual factors. These new factors would further strengthen the questionnaire construction, confirming achievement orientations dimensions that “penetrate” the notion of academic hardiness. Second order factor analysis, in contrast with the traditional research on instrument development which focused on primary scale factors, exams the possibility that various scales may be correlated^ii^ and that scales can be reduced further and explains more concisely by their underlying constructs (Gorsuch, 1983).

Reliability of the scales was examined using Cronbach’s alpha coefficient and Guttman-split-half. These procedures are the most commonly employed to estimate the internal consistency of test scores among several variables of personality. Pearson correlation was calculated to establish the convergent validity between the “Dimensions of Academic Hardiness Questionnaire” and late elementary school children’s coping strategies. In order to further investigate the convergent validity of the questionnaire in the frame of the theoretical convergence between the Academic Hardiness and Coping, we conducted one-way analysis of variance (ANOVA) to investigate the adoption of different coping strategies by children with low, medium and high scores in the “Dimensions of Academic Hardiness Questionnaire”.

## Results

### Confirmatory Factor Analysis

A CFA using items derived from the previous exploratory and confirmatory factor analyses (Kamtsios & Karagiannopoulou, 2013b) based upon data from 1264 late elementary school children was performed. ML estimation was employed to estimate the model. Factor loadings and error variances of the CFA are presented in Table 2. Examination of the fit indices indicated a good fit of the proposed model to the data. The chi-square ratio to degrees of freedom was 1496.79/568, more than the desired 2:1 ratio; however this was not unexpected due to the sample size (Floyd & Widaman, 1995; Jöreskog, 1969) and leads the researchers to the use of additional indices. The 9-factor model had good fit with the data as indicated by the following indices: CFI: .91, GFI: .91, AGFI: .90, RMR: .05, SRMR: .06, RMSEA: .05, NFI: .84, NNFI: .90.

**Table 2:** Factor Loadings and Error Variances of the “Dimensions of Academic Hardiness Questionnaire” for Late Elementary School Children

No	Item	Factor								
		1	2	3	4	5	6	7	8	9
1	I do my best at school so as my marks to be higher than those of my classmates.	.81 (.58)								
2	I do my best at schoolwork because I want not only to get a good mark but also to be among the best pupils of my class.	.82 (.57)								
3	I do my best at school in order to achieve the marks I want and to be better than my schoolmates	.73 (.68)								
4	I try to have good marks because I don’t want my friends to make fun of me.	.63 (.77)								
5	When I get a low mark I try to "unblock" and think rationally.		.49 (.87)							
6	When my performance at school in not good, I try to find ways to face the problem.		.45 (.89)							
7	Getting a low mark is something unpleasant, but I believe that if I try a lot I can make it.		.59 (.79)							
8	I try not only to calm down, when my performance at school is not so satisfying, but also to think about what I can do to improve it.		.60 (.79)							
9	I try to calm down and realize what went wrong in order to do something about that.		.57 (.81)							
10	If I get a low mark, I try to do something, in order to forget about what happened for a while before I make a decision about my next step.		.37 (.92)							
11	I do care about getting a good mark in order to make my parents feel pleased/satisfied.			.69 (.71)						
12	I do my best at schoolwork to prove to my parents that I can make it.			.73 (.67)						
13	Through my good performance I can prove to my teacher that I can make it at school.			.63 (.77)						
14	Having in mind that a possible failure in a test can disappoint my teacher, I do my best to avoid it.			.63 (.77)						
15	I try to be really attentive to my schoolwork as some of the subjects taught may be handful/useful later in my life.				.59 (.80)					
16	I believe that everything taught and learnt at school now can also be used in both secondary and high school.				.52 (.85)					
17	It is very important for me personally to get a good mark.				.52 (.85)					
18	I study my lessons carefully/thoroughly because the knowledge acquired can benefit my life in the long term.				.57 (.81)					
19	I make an effort for all lessons. All lessons taught can help me in my future life				.48 (.87)					
20	My concern about my parents’ potential reaction to a low mark makes me try harder					.55 (.83)				
21	My concern about my teacher’s reaction to a possible failure makes me study more					.60 (.80)				
22	I try not to get a low mark to avoid feeling disappointment and shame					.53 (.84)				
23	I do my homework because, if I get a low mark, I may feel bad/unpleasant					.62 (.78)				
24	A low mark makes me feel sad but if I study more, this is not going to happen again					.42 (.90)				
25	I spend time in after school activities (e.g. playing out with my friends) only after I have finished my school homework						.61 (.78)			
26	I try to finish my homework first before I spend time with my friends						.63 (.76)			
27	I do my homework first and then I play with my friends						.63 (.77)			
28	I find interest in my school subjects even though they may be difficult							.67 (.73)		
29	I find interest in the content even of those lessons considered as difficult							.67 (.72)		
30	I try my best, even at the difficult subjects/lessons, through daily revisions							.53 (.84)		
31	When I have difficulties with my schoolwork I ask for my parents’ help								.77 (.63)	
32	I ask for my parents’ help when I have questions/difficulties								.73 (.65)	
33	When I have difficulties, I prefer asking for an adult’s help								.63 (.77)	
34	I don’t feel disappointed when I get a low mark. On the contrary I try harder to improve myself									.60 (.79)
35	Failing a test doesn’t disappoint me, but it makes me try harder									.69 (.72)
36	Getting a low mark makes me try harder in order to get a higher one the next time									.52 (.85)

*Note.* Values in parentheses are error variances.

### Reliability Analysis

Alpha reliability provided measures of internal consistency. The alpha coefficient for the entire instrument was .91, which is high (Henson, 2001; Yang & Green, 2011). Alpha coefficients for the 9 factors ranged from .68 to .83. Split-half ranged from .61 to .81. Therefore, the factors of the questionnaire offered adequate psychometric properties for the evaluation of this construct (DeVellis, 2003). Table 3 presents the alpha coefficients and spit-half for the 9 factors as well as the means and standard deviations for the 9 scales.

**Table 3:** Number of Items, Means and % of Total Variance, Cronbach's α and Split-Half of the “Dimensions of Academic Hardiness Questionnaire” for Late Elementary School Children - Correlations Between the Factors

No	Factor	Number of items	*M* (*SD*)	% of total variance	Cronbach's α	Split-half	1	2	3	4	5	6	7	8	9
1	Commitment: comparing oneself with peers and acceptance from peers	4	2.93 (.68)	7.3	.83	.81		.20**	.59**	.18*	.45**	.15*	.20**	.27**	.07
2	Control-awareness: use of effective coping strategies	6	3.21 (.42)	7.1	.74	.70			.33**	.56**	.45**	.41**	.43**	.16*	.46**
3	Commitment: adults’ acceptance	4	3.15 (.50)	6.3	.75	.72				.31**	.54**	.24**	.30**	.24**	.16*
4	Commitment: knowledge utility	5	3.64 (.44)	6.3	.74	.73					.42**	.49**	.47**	.21**	.42**
5	Control-awareness: attempt to avoid unpleasant feelings	5	3.20 (.34)	6.1	.70	.69						.27**	.36**	.23**	.25**
6	Commitment: regulating priority to learning vs. enjoyment	3	3.45 (.63)	5.9	.68	.62							.41**	.16**	.38**
7	Challenge: dealing positively with hard subjects	3	3.15 (.30)	5.5	.66	.57								.16*	.39**
8	Commitment: looking for help contributing to learning	3	3.05 (.42)	5.3	.77	.66									.12*
9	Challenge: dealing with failure in a constructive way	3	3.30 (.40)	4.6	.64	.59									

*p < .05. **p < .01.

### Convergent Validity

**Figure 1:**
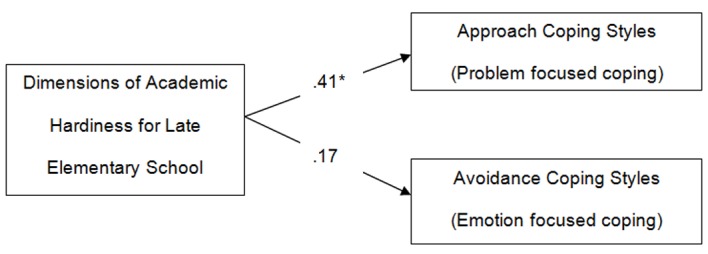
Correlations between the “Dimensions of Academic Hardiness Questionnaire” for late elementary school children and coping styles (approach and avoidance). *p < .05.

To provide preliminary estimates of convergent validity of the “Dimensions of Academic Hardiness Questionnaire” we examined the relationship of the questionnaire with the approach and avoidance coping strategies, measured by the “Athens Coping Scale Questionnaire” (Besevegis, 2001). This questionnaire was administered as a criterion validity measure to examine the relationship of the newly developed scale with children’s preferred coping strategies. Correlations between the “Dimensions of Academic Hardiness Questionnaire” and coping styles (approach and avoidance) are displayed in Figure 1. As anticipated, the total score of the “Dimensions of Academic Hardiness Questionnaire” was correlated positively and highly with the approach coping styles (problem focused coping styles).

Results from correlation analysis also revealed a statistical significance relation between the 9-factors of the “Dimensions of Academic Hardiness Questionnaire” with coping styles as family support, problem solving, assistance keeping and revision-reorganization (Table 4).

**Table 4:** Correlations Between the 9 Factors of the “Dimensions of Academic Hardiness Questionnaire” and the Coping Styles

No	Factor	1	2	3	4	5	6	7	8	9	10	11	12	13	14	15	16
1	Commitment: comparing oneself with peers and acceptance from peers		.14**	.57**	.13*	.43**	.10*	.15**	.20**	.03	.01	.09	.20**	.06	.07	-.03	.09
2	Control-awareness: use of effective coping strategies			.32**	.55**	.51**	.36**	.46**	.11**	.51**	.14**	.12*	-.08	.20**	.05	.14*	.36**
3	Commitment: adults' acceptance				.26**	.60**	.16**	.25**	.19**	.14**	-.005	.09	.13*	.08	.09	.002	.16*
4	Commitment: knowledge utility					.47**	.42**	.44**	.16*	.44**	.17*	-.04	-.14**	.19**	-.04	.08	.31**
5	Control-awareness: attempt to avoid unpleasant feelings						.27**	.37**	.15**	.28**	.007	.03	.08	.22**	.06	.05	.30**
6	Commitment: regulating priority to learning vs enjoyment							.34**	.07	.30**	.09	-.01	-.10*	.13**	-.03	.04	.24**
7	Challenge: dealing positively with hard subjects								.08	.38**	.15*	-.02	-.06	.12*	.06	.11*	.22**
8	Commitment: looking for help contributing to learning									.02	.34**	.08	.03	.11	.05	.15**	.12**
9	Challenge: dealing with failure in a constructive way										.14*	-.04	.-17**	.07	.-09	.08	.26**
10	Family support											19*	.-02	.31**	.15**	.43**	.39**
11	Avoidance												.39**	.15**	.23**	.28**	.22**
12	Giving up													.009	.32**	.16*	.-08
13	Problem solving														.25**	.25**	.44**
14	Isolation															.34**	.12*
15	Assistance keeping																.29**
16	Revision-reorganization																

*p < .05. **p < .01.

Also, as expected, one-way ANOVAs’ results revealed that children with high scores in the “Dimensions of Academic Hardiness Questionnaire” used approach coping strategies in order to cope effectively with a school failure such as problem solving and revision-reorganization (Table 5). Both results (correlation analysis and one-way ANOVA analysis) provided support for the convergent validity of the “Dimensions of Academic Hardiness Questionnaire” for late elementary school children”.

**Table 5:** One-Way ANOVAs’ Results for the Relation Between Different Scores of the Dimensions of Academic Hardiness and Coping Strategies.

Coping strategy	Dimensions of Academic Hardiness			*F*	*p*
	Low	Medium	High		
	*M* (*SD*)	*M* (*SD*)	*M* (*SD*)		
Family support	2.89 (.84)	2.97 (.80)	3.04 (.80)	1.228	.294
Avoidance	2.40 (.65)	2.42 (.68)	2.55 (.64)	1.459	.234
Giving up	1.79 (.56)	1.86 (.47)	1.87 (.58)	.871	.420
Problem solving	3.03 (.58)	3.11 (.47)	3.31 (.89)	5.967*	.003
Isolation	1.85 (.64)	1.79 (.54)	1.98 (.67)	2.222	.110
Assistance seeking	2.35 (.68)	2.37 (.67)	2.50 (.79)	1.240	.291
Revision-reorganization	3.17 (.63)	3.33 (.49)	3.53 (.45)	3.254*	.000

### Second Order Factor Analysis

**Figure 2:**
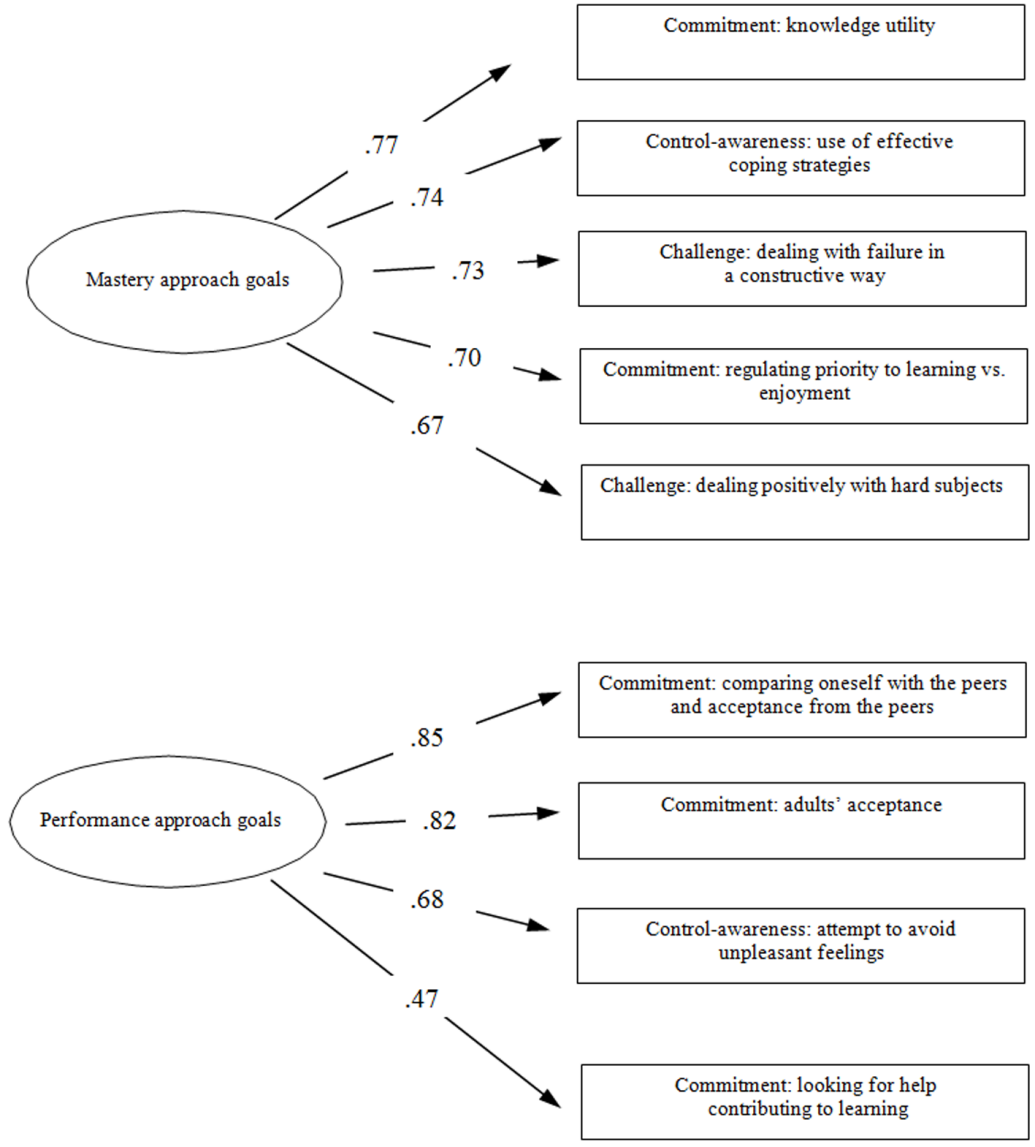
Second order factor analysis results.

A second order principal component factor analysis with varimax rotation was computed on the nine scales identified by the CFA. A number of procedures were used to determine the number of factors to extract: examination of the scree plot (Cattell, 1966), eigenvalues greater than one (Kaiser, 1970) and magnitude of item loadings. The scree-plot indicated the presence of two second order factors. A two-factor model was specified. The nine scales of the “Dimensions of Academic Hardiness Questionnaire” were explained by two factors (Figure 2). The first factor, which was labeled task orientation, included five of the nine scales composed by children’s mastery goals, whereas the second factor, which was labeled performance orientation, included four of the nine factors composed by children’s performance goals.

## Discussion

The study reports the validation of an instrument to measure dimensions of academic hardiness for late elementary school children. The result is an instrument with acceptable reliability that is appropriate for use in late elementary school children populations. Multiple fit indices support that the 9-factor model had a good fit to the data. The acceptance to high level of internal consistency, based on the total sample, indicates that the questionnaire and its subscales are reliable and can be used in late elementary school children. The study also provided preliminary evidence of convergent validity for the “Dimensions of Academic Hardiness” scores. Results indicated a high correlation between the “Dimensions of Academic Hardiness” scores and approach coping styles (problem focused coping strategies). The relation between the dimensions of academic hardiness and coping is consistent with research suggesting that high hardy people cope with stresses and stressful circumstances by the use of approach coping styles (Chan, 2000; Clarke, 1995; Florian et al., 1995; Lambert et al., 2003). Results from the second order factor analysis support the research hypothesis that there is a relation between the dimensions of academic hardiness and achievement goal orientations in student learning, as children’s orientations to mastery or performance goals are noted. This section below will explain these results; will suggest directions for future research, limitations and will discuss potential implications of these findings for school settings.

### Structural, Convergent and Content Validity of the Scale

The nine factors which resulted from the CFA reflect the different ways in which late elementary school children try to manage school failure. The positive correlations between the factors (Table 3) imply their complementarity; namely children’s way to cope with school failure. According to the results of the study this could be an important finding as problems in the school environment, because of a failure, are multifaceted and children use different ways and strategies to cope with them. The statistically significant correlations between the “Dimensions of Academic Hardiness” and the “approach oriented” coping styles (Figure 1) support the convergent validity of the scale. The findings of the study shed light on Benishek’s, Feldman’s, Shipon’s, Mecham’s, and Lopez’s (2005) suggestions for further exploration of academic hardiness as there are additional aspects of the construct that are yet to be identified and Cole et al. (2004) hypothesis for the interaction between academic hardiness and academic motivation. In particular, the results of the study set off dimensions of academic hardiness, dimensions of commitment control and challenge, which have not been reported in the existing literature. These theoretical dimensions are conceptually categorized in the three academic hardiness attitudes (commitment, control, challenge). They provide a framework for understanding and comprehension of children’s reaction to school failure. Also the results point out the relation between academic hardiness and learning motivation. To our knowledge, this is the first study that provides statistical support for the relation between the dimensions of academic hardiness and academic motivation (Pintrich, 2004).

### Contribution of the Present Study Concerning Control

The results of the study enrich the notion of academic hardiness in late elementary school children. In particular, from the second and the fifth factor (control-awareness: use of effective coping strategies and control-awareness: attempt to avoid unpleasant feelings) the role of awareness and the role of children’s previous experiences are distinguished. Specifically, according to the conceptual content of the second factor, children’s “detachment” from the problem (the school failure) is distinguished, together with children’s volition to control their emotions in order to cope with the difficult situation (failure). Children’s ability to “detach” from the problem - the extent to which there is a sense of a perceptually distinct object being “observed” (Lambie, 2008) - through the control of negative emotions is particularly important. Unpleasant emotions transmit a negative relation between achievement goals in children learning and the use of strategies with regard to their achievement (Linnenbrink, Ryan, & Pintrich, 1999). This finding, with regard to children’s detachment from the problem could be important. If children deluge by the negative/unpleasant emotions are less likely to use deeper learning strategies and are less likely to elaborate properly their knowledge. As a result they have less control over the situation which consequently may lead to negative affect (Dweck, 2000).

The results of the present study strengthen the significance of control in children’s learning. The importance of their past experiences for the regulation of their behavior is distinguished as children, determine their behavior according to their previous emotional experiences, (e.g. fifth factor, question No 22: “I try not to get a low mark to avoid feeling disappointment and shame”; question No 20: “My concern about my parents’ potential reaction to a low mark makes me study more”). This finding, concerning children’s awareness, theoretically strengthens the significance of control. On the basis of previous experiences children try to avoid the unpleasant emotions which proceed due to the school failure. These emotions, referred to their previous experiences, have created emotional memories (Eccles & Wigfield, 2002). The results of the study point out that previous negative experiences concern the unpleasant/negative emotions and the negative consequences after a school failure. Children’s awareness for the previous negative experience is activated and can lead to their further engagement. Children’s choice is to try harder so as not to experience again the negative emotions that follow a school failure. Children's control increases the possibility for them to cope with the experience as a result of their action mainly and not as an event that they do not expected or as something excessively. Children can recognize and appraise both the causes and the consequences of their failure. They can also acknowledge the unpleasant/negative feelings they had experienced e.g. the low grade (Kamtsios & Karagiannopoulou, 2013a). The two dimensions of awareness (control-awareness: use of effective coping strategies and control-awareness: attempt to avoid unpleasant feelings) are consistent with previous research mentioning that awareness crucially allows emotional responses to be inhibited: such inhibition is necessary for truly rational action selection (Lambie, 2008).

### Contribution of the Present Study Concerning Commitment

The results of the present study, with regard to commitment, imply that commitment enriched with achievement goal orientations. These motivational orientations had not been determined in the initial approach of academic hardiness theory (Benishek et al., 2005) and may have an influence on how information (school failure) is sought and how information is evaluated with respect to goal attainment. Task and performance orientations affect the way late elementary school children cope with the school failure (e.g. commitment: knowledge utility, commitment: comparing oneself with peers and acceptance from peers). Children’s future expectations also shape the behavior they choose to have in the present in order to cope with school failure (e.g. commitment: knowledge utility).

### Contribution of the Present Study Concerning Challenge

With regard to challenge, the results of the study are in line with hardiness theory (Kobasa, 1979; Maddi, 2006) and academic hardiness theory (Benishek et al., 2005). The present study implies that challenge concerns children’s belief and appraisal of a particular stressor as an opportunity and motivation for learning, instead of thinking of it as something threatening (Kamtsios & Karagiannopoulou, 2013a, 2013b) (e.g. challenge: dealing positively with hard subjects; challenge: dealing with the failure in a constructive way). In such a way children can be taught by their errors and they can develop learning strategies in order to confront future school and learning demands.

The theoretical dimensions of academic hardiness revealed in the present study are important for interpretation and understanding of children’s behavior in a matter of school failure (low grade in a test). The dimensions of academic hardiness may be particularly significant for children’s ability to cope with difficulties in school environment. As seen in Figure 1 the dimensions of academic hardiness were strongly correlated with problem focused coping strategies. The finding confirm research hypothesis and suggest that the dimensions of academic hardiness help late elementary school children to cope effectively with a school failure. Similar support comes from ANOVA results (Table 4). These results are consistent with past research evidence that indicate a strong relationship between approach coping styles and high hardy persons (Green et al., 2007; Maddi, 2006; Sheard, 2009) or academic hardy undergraduates (Hystad et al., 2009; Lifton et al., 2006). It can be suggested that the dimensions of academic hardiness have a buffering effect against school and learning related stressful events, which influences the type of particular coping strategies late elementary school children utilize.

The results of the study revealed that the dimensions of commitment, control and challenge may have a buffering effect against school and learning related stress. From the conceptual meaning of the nine factors of the questionnaire we can hypothesize that the mechanism whereby this beneficial effect takes place seems to involve the tendency of high academic hardiness children to: (a) View school life events (e.g. failure) as less stressful (e.g. factor 7 & 9), (2) Cope effectively with these events (e.g. factor 2), (3) Avoid excessive psychological arousal and be aware of their feelings (factor 5) and (4) Pursue positive practices (e.g. factor 2 & 6). This mechanism is similar to the original hardiness theory (Maddi, 2006) whereby hardiness constitutes positivity and resiliency in meeting life’s changes (Maddi, 2005).

In relation to the original hardiness theory (Kobasa, 1979; Maddi, 2006) and academic hardiness theory (Benishek et al., 2005), the newly developed and evaluated questionnaire, measuring academic hardiness dimensions for late elementary school children, describes academic hardiness dimensions as a children-personality variable which has cognitive, behavioral and emotional aspects (Kamtsios & Karagiannopoulou, 2013b). The relation between academic hardiness dimensions and motivation is also explored. Motivation to learn influences the decision-making processes determining the direction, focus and level of effort children will apply to a learning-educational context (Pintrich, 2004).

### Implementation

From a didactic point of view these findings are particularly interesting. High academic hardiness children appear more aware for the reason they experience the unpleasant situation. Children’s control-awareness increases the probability for them to face the experience as a result of their action mainly and not as an event that they did not expect or as something excessive. Children can also recognize and appraise both the causes and the consequences of their failure. They can also acknowledge the unpleasant/negative feelings that they experienced because of the low grade (Kamtsios & Karagiannopoulou, 2013b). Also high academic hardiness children use coping strategies that are most active and problem focused, whereas those who are low hardy tend to cope by avoiding or denying threat.

### Study Limitations and Further Research Suggestions

A number of study limitations and directions for future work warrant comment. The current study used fifth and sixth graders. Future studies should include older children. Although the current study has provided initial evidence of convergent validity for the “Dimensions of Academic Hardiness” questionnaire, further research to establish the validity of the questionnaire is very much needed. The results of the study may hold important practical implications for educators. The more educators learn about their students, the more able they will be to meet the needs of their students. Furthermore in terms of future research an important question is: are the dimensions of academic hardiness a stable personality trait or are they a personal resource that can be modified by the experience of everyday school events? Future studies should also investigate whether the results in this study emerge as a consistent finding or as an artifact of the present sample. The researchers are encouraged to proceed with applying the scale to other countries and educational settings and addressing further validity issues, such as its invariance among genders, educational level (elementary school, high school, and senior high school), cultures and contexts. Such studies can stimulate research because their results can be directly compared, enriching our knowledge of “Academic Hardiness Dimensions”, as a strong predictor of academic achievement, with other variables from the field of educational psychology.

## References

[r1] Amponi-Tsoura, L. (2006). *Stress and coping in the school age*. Paper presented at the 27th International Conference of the Stress and Anxiety Research Society, Crete, Greece.

[r2] Argyropoulou E. P. Sidiropoulou-Dimakakou D. Besevegis E. G. (2007). Generalized self-efficacy, coping, career indecision, and vocational choices of senior high school students in Greece: Implications for career guidance practitioners. Journal of Career Development, 33, 316–337. 10.1177/0894845307300412

[r3] Benishek L. A. Feldman J. M. Shipon R. W. Mecham S. D. Lopez F. G. (2005). Development and evaluation of the Revised Academic Hardiness Scale. Journal of Career Assessment, 13, 59–76. 10.1177/1069072704270274

[r4] Benishek L. A. Lopez F. G. (2001). Development and initial validation of a measure of academic hardiness. Journal of Career Assessment, 9, 333–352. 10.1177/106907270100900402

[r5] Bentler, P. M. (1995). *EQS structural equations program manual.* Encino, CA, USA: Multivariate Software, Inc.

[r6] Besevegis, E. (1996). *Facing daily stressful situations: A new coping scale*. Paper presented at the 5th Greek Congress of Psychological Research, Patra, Greece.

[r7] Besevegis, E. (2001). Stress, stressful situations and childrens’ and adolescents’ coping strategies. In H. Vasilaki, S. Triliva, & E. Besevegis (Eds.), *Stress, anxiety and coping* (pp. 29-60). Athens, Greece: Ellinika Grammata.

[r8] Brown, M. W., & Cudeck, R. (1993). Alternative ways of assessing model fit. In K. A. Bollen & J. S. Long (Eds.), *Testing structural equation models* (pp. 136-162). Newbury Park, CA, USA: Sage.

[r9] Cantoni E. Ronchetti E. (2006). A robust approach for skewed and heavy-tailed out-comes in the analysis of health care expenditures. Journal of Health Economics, 25(2), 198–213. 10.1016/j.jhealeco.2005.04.010 16413941

[r10] Cattell R. B. (1966). The Scree Test for the number of factors. Multivariate Behavioral Research, 1, 245–276. 10.1207/s15327906mbr0102_10 26828106

[r11] Chan D. W. (2000). Dimensionality of hardiness and its role in the stress-distress relationship among Chinese adolescents in Hong Kong. Journal of Youth and Adolescence, 29, 147–161. 10.1023/A:1005100531194

[r12] Clarke D. E. (1995). Vulnerability to stress as a function of age, sex, locus of control, hardiness, and Type A personality. Social Behavior and Personality, 23, 285–286. 10.2224/sbp.1995.23.3.285

[r13] Cole M. Field H. Harris S. (2004). Student learning motivation and psychological hardiness: Interactive effects on students’ reaction to a management class. Academy of Management Learning & Education, 3(1), 64–85. 10.5465/AMLE.2004.12436819

[r14] Compas B. E. Connor-Smith J. K. Saltzman H. Thomsen A. H. Wadsworth M. E. (2001). Coping with stress during childhood and adolescence: Problems, progress, and potential in theory and research. Psychological Bulletin, 127(1), 87–127. 10.1037/0033-2909.127.1.87 11271757

[r15] DeVellis, R. F. (2003). *Scale development: Theory and applications* (2nd ed.). Thousand Oaks, CA, USA: Sage.

[r16] Dweck, C. S. (2000). *Self-theories: Their role in motivation, personality, and development*. Philadelphia, PA, USA: The Psychology Press.

[r17] Eccles J. S. Wigfield A. (2002). Motivational beliefs, values, and goals. Annual Review of Psychology, 53, 109–132. 10.1146/annurev.psych.53.100901.135153 11752481

[r18] Florian V. Mikulincer M. Taubman O. (1995). Does hardiness contribute to mental health during a stressful real-life situation? The roles of appraisal and coping. Journal of Personality and Social Psychology, 68, 687–695. 10.1037/0022-3514.68.4.687 7738771

[r19] Floyd F. J. Widaman K. F. (1995). Factor analysis in the development and refinement of clinical assessment instruments. Psychological Assessment, 7, 286–299. 10.1037/1040-3590.7.3.286

[r20] Giavrimis P. Konstantinou E. Hatzichristou C. (2003). Dimensions of immigrant students’ adaptation in the Greek schools: Self-concept and coping strategies. Intercultural Education, 14(4), 423–434. 10.1080/1467598032000139859

[r21] Golightly, T. (2007). *Defining the components of academic self efficacy in Navajo Indian high school students* (Unpublished doctoral dissertation). Brigham Young University, Provo, UT, USA.

[r22] Gorsuch, P. L. (1983). *Factor analysis* (2nd ed.). Hillsdale, NJ, USA: Lawrence Erlbaum.

[r23] Green S. Grant A. Rynsaard T. L. (2007). Evidence-based life coaching for senior high school students: Building hardiness and hope. International Coaching Psychology Review, 2(1), 24–32.

[r24] Henson R. K. (2001). Understanding internal consistency reliability estimates: A conceptual primer on coefficient alpha. Measurement and Evaluation in Counseling and Development, 34(3), 177–189.

[r25] Hu L.-t. Bentler P. M. (1998). Fit indices in covariance structure modeling: Sensitivity to underparameterized model misspecification. Psychological Methods, 3, 424–453. 10.1037/1082-989X.3.4.424

[r26] Hu L.-t. Bentler P. M. (1999). Cutoff criteria for fit indexes in covariance structure analysis: Conventional criteria versus new alternatives. Structural Equation Modeling, 6, 1–55. 10.1080/10705519909540118

[r27] Hystad S. Eid J. Laberg J. Johnsen B. Bartone P. (2009). Academic stress and health: Exploring the moderating role of personality hardiness. Scandinavian Journal of Educational Research, 53(5), 421–429. 10.1080/00313830903180349

[r28] Jöreskog K. G. (1969). A general approach to confirmatory maximum likelihood factor analysis. Psychometrika, 34, 183–202. 10.1007/BF02289343

[r29] Kaiser H. F. (1970). A second generation little jiffy. Psychometrika, 35, 401–415. 10.1007/BF02291817

[r30] Kamtsios S. Diggelidis N. (2008). Daily stress symptoms, sources of stress and stages of change for stress management in primary and secondary school children. Inquires in Sport and Physical Education, 6(3), 257–269.

[r31] Kamtsios S. Karagiannopoulou E. (2011). Psychometric characteristics of the “Academic Hardiness Scale” in a Greek sample: A pilot study. Scientific Annals of the Department of Psychology Aristoteleion University of Thessaloniki, 9, 67–88.

[r32] Kamtsios S. Karagiannopoulou E. (2013 a). Conceptualizing students’ academic hardiness dimensions: A qualitative study. European Journal of Psychology of Education, 28(3), 807–823. 10.1007/s10212-012-0141-6

[r33] Kamtsios S. Karagiannopoulou E. (2013 b). The development of a questionnaire on academic hardiness for late elementary school children. International Journal of Educational Research, 58, 69–78. 10.1016/j.ijer.2012.12.001

[r34] Karimi A. Venkatesan S. (2009). Mathematics anxiety, mathematics performance and academic hardiness in high school students. International Journal of Educational Sciences, 1(1), 33–37.

[r35] Kinder, R. (2008). *Development and validation of the students’ activation measure* (Unpublished doctoral dissertation). Vanderbilt University, Nashville, TN, USA.

[r36] Kline, P. (2000). *The handbook of psychological testing* (2nd ed.). London, United Kingdom: Routledge.

[r37] Kline, R. B. (1998). *Principles and practice of structural equation modelling*. New York, NY, USA: The Guillford Press.

[r38] Kobasa S. C. (1979). Stressful life events, personality, and health: An inquiry into hardiness. Journal of Personality and Social Psychology, 37, 1–11. 10.1037/0022-3514.37.1.1 458548

[r39] Kranzler J. H. Keith T. Z. (1999). Independent confirmatory factor analysis of the cognitive assessment system (CAS): What does the CAS measure? School Psychology Review, 28, 117–144.

[r40] Lambert V. A. Lambert C. E. Jr. Yamase H. (2003). Psychological hardiness, workplace stress and related stress reduction strategies. Nursing & Health Sciences, 5(2), 181–184. 10.1046/j.1442-2018.2003.00150.x 12709174

[r41] Lambie J. A. (2008). On the irrationality of emotion and the rationality of awareness. Consciousness and Cognition, 17, 946–971. 10.1016/j.concog.2007.03.005 17451970

[r42] Lazarus, R. S. (1999). *Stress and emotion: A new synthesis.* New York, NY, USA: Springer

[r43] Lazarus, R. S., & Folkman, S. (1984). *Stress, appraisal, and coping.* New York, NY, USA: Springer.

[r44] Lifton D. Seay S. McCarly N. Olive-Taylor R. Seeger R. Bighee D. (2006). Correlating hardiness with graduation persistence. Academic Exchange Quarterly, 10, 277–282.

[r45] Linnenbrink E. A. Ryan A. M. Pintrich P. R. (1999). The role of goals and affect in working memory functioning. Learning and Individual Differences, 11, 213–230. 10.1016/S1041-6080(00)80006-0

[r46] Maddi S. R. (2005). On hardiness and other pathways to resilience. American Psychologist, 60(3), 261–262. 10.1037/0003-066X.60.3.261 15796684

[r47] Maddi S. R. (2006). Hardiness: The courage to grow from stresses. The Journal of Positive Psychology, 1(3), 160–168. 10.1080/17439760600619609

[r48] Maddi S. Harvey R. Khoshaba D. Fazel M. Resurreccion N. (2009). The personality construct of hardiness, IV: Expressed in positive cognitions and emotions concerning oneself and developmentally relevant activities. Journal of Humanistic Psychology, 49(3), 292–305. 10.1177/0022167809331860

[r49] Maddi S. R. Harvey R. H. Khoshaba D. M. Fazel M. Resurreccion N. (2012). The relationship of hardiness and some other relevant variables to college performance. Journal of Humanistic Psychology, 52(2), 190–205. 10.1177/0022167811422497

[r50] Maddi, S., & Kobasa, S. (1984). *The hardy executive: Health under stress*. Chicago, IL, USA: Dorsey.

[r51] Mathis M. Lecci L. (1999). Hardiness and college adjustment: Identifying students in need of services. Journal of College Student Development, 40(3), 305–309.

[r52] Morrissey C. Hannah T. E. (1987). Measurement of psychological hardiness in adolescents. The Journal of Genetic Psychology, 148, 393–395. 10.1080/00221325.1987.9914569 3655759

[r53] Nelms B. C. (1999). Stress and school: Helping children cope. Journal of Pediatric Health Care, 13(5), 209–210. 10.1016/S0891-5245(99)90000-7 10776194

[r54] Olsson U. H. Foss T. Troye S. V. Howell R. D. (2000). The performance of ML, GLS, and WLS estimation in structural equation modeling under conditions of misspecification and nonnormality. Structural Equation Modeling, 7, 557–595. 10.1207/S15328007SEM0704_3

[r55] Paleologou A.-M. Dellaporta A. (2009). Hardiness vs. alienation personality construct essentially explains burnout proclivity and erroneous computer entry problems in rural Hellenic hospital labs. International Journal of Social, Behavioral, Educational, Economic, Business and Industrial Engineering, 3, 282–297.

[r56] Pincus D. B. Friedman A. G. (2004). Improving children’s coping with everyday stress: Transporting treatment interventions to the school setting. Clinical Child and Family Psychology Review, 7(4), 223–240. 10.1007/s10567-004-6087-8 15648277

[r57] Pintrich P. R. (2004). A conceptual framework for assessing motivation and self-regulated learning in college students. Educational Psychology Review, 16, 385–407. 10.1007/s10648-004-0006-x

[r58] Sheard M. (2009). Hardiness, commitment, gender, and age differentiate university academic performance. British Journal of Educational Psychology, 79(1), 189–204. 10.1348/000709908X304406 18466672

[r59] Tabachnick, B. E., & Fidell, L. S. (1996). *Using multivariate statistics*. New York, NY, USA: Harper Collins.

[r60] Tanaka, J. S. (1993). Multifaceted conceptions of fit in structural equation models. In K. A. Bollen & J. S. Long (Eds.), *Testing structural equation models* (pp. 10-39). Newbury Park, CA, USA: Sage.

[r61] Yang Y. Green S. B. (2011). Coefficient alpha: A reliability coefficient for the 21st century? Journal of Psychoeducational Assessment, 29, 377–392. 10.1177/0734282911406668

